# Stick-Slip Vibration Suppression in Drill String Using Observer-Based LQG Controller

**DOI:** 10.3390/s22165979

**Published:** 2022-08-10

**Authors:** Rami Riane, Mohamed Zinelabidine Doghmane, Madjid Kidouche, Kong Fah Tee, Sofiane Djezzar

**Affiliations:** 1 Laboratory of Applied Automatic, Department of Automation, Faculty of Hydrocarbons and Chemistry, University M’hamed Bougara of Boumerdes, Boumerdes 35000, Algeria; 2 Reservoirs’ Evaluation Department, DOE, Exploration Division, Sonatrach, Hassi Messaoud 30500, Algeria; 3 School of Engineering, University of Greenwich, Central Avenue, Chatham Maritime, Kent ME4 4TB, UK; 4 Petroleum Engineering Department, University of North Dakota (UND), Grand Forks, ND 58202, USA

**Keywords:** LQG, observer-based controller, rotary drilling systems, drill string, SimScape/Matlab environment, stick-slip vibrations, torque on bit

## Abstract

Hydrocarbon exploration and production activities are guaranteed through various operations including the drilling process, which is realized by using rotary drilling systems. The process involves crushing the rock by rotating the drill bit along a drill string to create a borehole. However, during this operation, violent vibrations can occur at the level of the drill string due to its random interaction with the rocks. According to their axes of occurrence, there are three types of vibrations: axial, lateral, and torsional, where the relentless status of the torsional vibrations is terminologically known as the stick-slip phenomenon. Such a phenomenon can lead to increased fatigue of the drill string and cause its abortive fracture, in addition to reducing the efficiency of the drilling process and consequently making the exploration and production operations relatively expensive. Thus, the main objective of this paper is to eliminate the severe stick-slip vibrations that appear along the drill string of the rotary drilling system according to the LQG observer-based controller approach. The rock–bit interaction term is highly nonlinear, and the bit rotational velocity is unmeasurable; an observer was first designed to estimate the unknown inputs of the model, and then the controller was implemented in the drill string model with 10 degrees of freedom. The estimation process was essentially based on surface measurements, namely, the current and rotational velocity of the top drive. Thereafter, the performance of the proposed observer-based LQG controller was tested for different simulation scenarios in a SimScape/Matlab environment, for which the controller demonstrated good robustness in suppressing the severe stick-slip vibrations. Furthermore, the simulation and experimental results were compared to other controllers designed for the same model; the proposed observer-based LQG controller showed better performance, and it was less sensitive to structured disturbances than H∞. Thence, it is highly recommended to use the proposed approach in smart rotary drilling systems.

## 1. Introduction

The drilling process is the set of operations allowing the digging of holes in the subsurface to arrive at the targeted reservoirs. Thus, the performance of hydrocarbon exploration and production depends on the drilling process systems, for which the most common in the oil industry is called the rotary drilling system [1]. Since this process constitutes the main and the essential part of the total cost of an oil well, its efficiency, reliability, and performance are one of the most important aspects of deep well drilling. Nevertheless, one of the main restrictive factors of these drilling aspects is the presence of severe stick-slip vibrations in the drill string; these vibrations reduce the quality of the drilling process, causing premature wear of the drilling equipment and, in extreme cases, inducing the breakage of the drill string and halting the drilling operations totally [2]. For these reasons, the attenuation of such vibrations to improve the drilling performance received increased attention from researchers in the last few years, as well as great economic interest from the petroleum industries. To this end, this research work is dedicated to implementing a system for the detection and control of the most commonly occurring vibrations known as the stick-slip phenomenon [3]. The latter mainly encompasses the severe case of the torsional vibration; it is caused by the elasticity of the drill string and the discontinuous nature of the torques acting along with it [4]. Because the use of a one-piece drilling tool with fixed bits can accentuate this phenomenon, the drill string can induce the stick-slip vibrations additionally to the drill bit [5]. This is mainly due to the fact that the drill string can be in contact with the borehole even if the diameter of the well is much larger than the diameter of the drill pipes. There are principally two reasons why this contact occurs. The first is that, for a long drill string, it will get a bent shape in the well due to its weight, the imbalance, and the centrifuge forces effects. The second reason is that, in the last few years, it has become more common to drill deviated wells, where the contact force between the drill string and the wellbore becomes sufficiently large; consequently, the stick-slip can occur anywhere along the drill string.

Due to the effects of torsional vibrations during drilling, including the stick-slip phenomenon, many methods and systems have been proposed over the last few years to eliminate them [6]. From the practical side, drilling operators have developed their own practices by reducing the weight on the bit and/or increasing the speed of the top drive. Even though these manipulations are a temporal solution, they remain manual operations limited in reliability, precision, and implementation speed [7]. From the technological side, smarter and more reliable automatic systems have been set up by oil service companies as a plug-in control software solution to eliminate torsional vibrations from the drill string, while maintaining a good rate of penetration [8,9,10]. From the academic side, several papers have developed different control strategies to eliminate torsional vibrations in the drill string [11], most of which used models with two degrees of freedom for the construction of the controller [3,12,13,14,15]. This model was proven simple, practical, and sufficient by the results given by Detournay and Defourny [5], but it was hypothetically supposed that the stick-slip occurs at low frequencies, which is not always true. Later on, Fu et al. [16] used a model with several degrees of freedom, while Abdulgalil and Siguerdidjane [10] discussed other types of nonlinearities. They assumed that the angular velocity of the drill bit is measurable, which requires the implementation of real-time measurement while drilling (MWD) tools; this is not always practically possible in all rigs [11]. Therefore, recent works introduced estimators of speed and/or torque on the bit to control it [16,17]. Unlike the previously cited papers that used the speed or the torque of the top drive as a control input for the attenuation of the torsional vibrations in the drill string, Cai et al. [18] adopted a new approach based on Wob (weight on bit) as the control input, which gave good results in experiments, but it is difficult to be realized in the field due to the slow dynamic of Wob manipulations [14,19]. Moreover, Trujillo-Franco et al. proposed [20] a time-domain algebraic identification scheme for online modal parameter estimation of flexible structures under linear and nonlinear vibrations; the obtained numerical and experimental results demonstrated good performance in suppressing the resonant excitations associated with the first mode of vibration. However, the effect of time delay was not considered for real-time control, in addition to not investigating the effectiveness of the approach for the active vibration damping, which is the case of the studied system in this paper. Baz et al. (1992) [21] demonstrated experimentally that the combination of independent modal space control (IMSC) with positive position feedback (PPF) provided superior vibration suppression performance to pseudo-inverse (PI) and modified independent model space control (MIMSC). The experiments were conducted on the beam actuator system, which is similar to the system considered in our paper. However, unlike our proposed approach, the algorithm proposed by Baz et al. is effective only for structural vibration with time-sharing capabilities. The condition is not satisfied for rotary drilling systems; thus, our proposed approach can be useful in this case.

Serrarens et al. proposed [22] the use of H∞ to suppress stick-slip vibrations in oil well drill strings; they adopted a linear approach where the transient behavior was improved using a PD-like control system. Even though this linear approach demonstrated good robustness in minimizing the nonlinear dynamics of the stick-slip vibrations, its main practical limitation is that the considered model was simplified using two degrees of freedom. Later on, Tian et al. [23] considered a four-degree-of-freedom lumped parameter model and proposed two types of sliding mode controllers to improve the mitigation of stick-slip vibrations; the main advantage of this study is that it considered the complex dynamics of the drill string. However, the chattering phenomenon of the sliding controller is still a challenging aspect when applying this approach in the petroleum drilling field. More recently, Riane et al. [24] considered a 10-degree model for the drill string and proposed the use of H∞ as an observer-based controller; the system was tested under different scenarios, where the obtained results demonstrated better performance and accuracy than those in [22] because more drill string complexity was studied. Even though the proposed H∞ demonstrated better performance in suppressing the stick-slip under unstructured perturbations than LQG, the latter could provide improvements under high-frequency stick-slip vibrations with structured perturbations that appear more often during drilling. Therefore, the main contribution of this paper is the suppression improvement of high-frequency stick-slip vibrations under structured perturbations in drill string of rotary drilling systems.

The work carried out in this manuscript falls within the framework of drill bit speed estimation and control based on surface drilling measurements using the LQG observer-based controller. The obtained results were compared to H∞ observer-based control in order to highlight its importance. This will provide a robust strategy for real-time detection and elimination of the severe stick-slip vibrations along the drill string under both structured and unstructured perturbations.

## 2. Vibrations in Drill String

The particularly worrisome regime during the drilling operation is the self-sustaining regime. In such a case, a constant disturbance appears such that the drill string enters an unpredictable vibrating regime that expands into a stable limit cycle [25]. Thence, the vibration itself generates its excitement. Moreover, the limit cycle corresponds to the resonance of the structure, which oscillates at a frequency close, but not equal, to its natural frequencies [26]. The torsional vibrations are the most appealing type of vibrations in the drill string [27], where its limit cycle lead generally to severe dynamics; accordingly, the severity is quantitatively evaluated in the next subsection.

### 2.1. Torsional Vibration Severity

Since the stick-slip is the extreme form of torsional vibrations, its appearance causes a periodic stop of the drill bit. In the course of this periodic stop, the drill string, driven in rotation from the surface, is twisted by virtue of the pipe’s elasticity [4]. Then, the drill bit is loosened up as soon as the bottom torque is greater than the torque of static friction [10]. The drill string relaxation then causes a strong acceleration of the bottom hole assembly (BHA), which exceeds by several times the speed on the surface [27]. Although the physical causes of the stick-slip are still only roughly understood, most researchers agree on the fact that, during this stick-slip, dynamic variations are accompanied by a variation in the resistive torque above the drill bit [28]. This is principally caused by the difference between the static and dynamic friction coefficients between the polycrystalline diamond compact (PDC) bit cutting edge and the formation to be drilled [6,29,30,31]. It has been deduced from field observations that the torsional vibrations develop especially when using PDC bits, which consume more torque than the tri-cone bits [26]. Such a dynamic has been studied by many researchers through a torsion pendulum equivalent system; however, its equations of motion are valid only for pure torsional vibrations [1,8,13,29,31]. Stick-slip is a self-sustaining phenomenon; it is for that reason that a sophisticated solution should be developed for detecting the stick-slip vibration, reducing its severity, and consequently optimizing the real-time drilling parameters [29]. To quantitatively evaluate the severity of the stick-slip vibrations (SS%), a new equation is proposed in this study as given by Equation (1) [24].

(1) $$SS=\frac{\Delta Speed}{Mean\left(speed\right)}\%.$$

The change in the bit rotational speed is normalized by its average, and the *SS%* is classified into four severity levels, as given in Table 1 [24]. This work constitutes a contribution to the detection and elimination of levels 2 and 3 of the vibrations using the observer-based LQG controller [15].

### 2.2. Drill String Model under Torsional Vibrations

**Figure 1:**
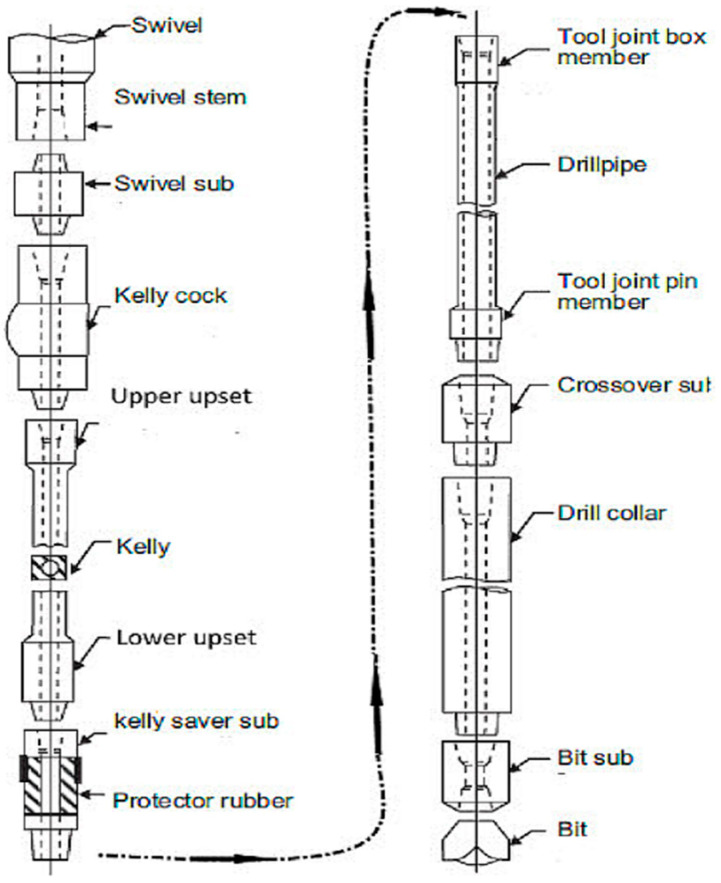
Drill string composition in a rotary drilling system [24].

**Figure 2:**
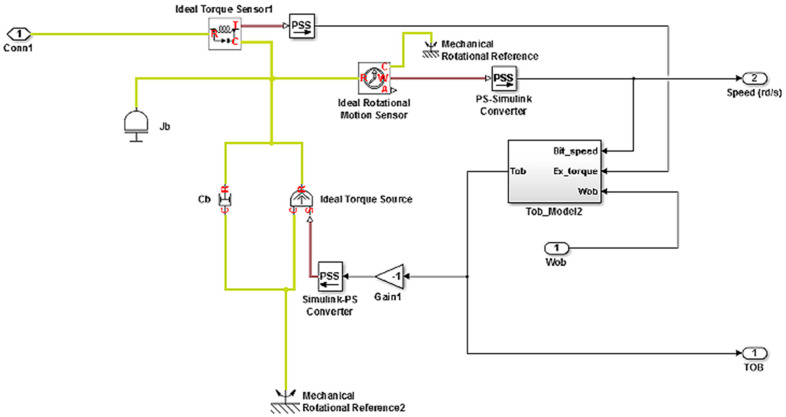
Rock–bit interaction block in SimScape environment [24].

**Figure 3:**
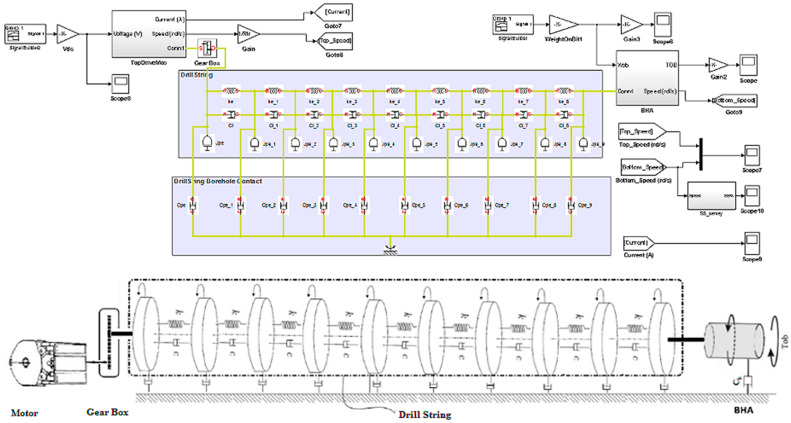
SimScape model of the drilling system with 10 degrees of freedom [24].

The mathematical model of the drilling system can be derived by representing the behavior of the system as the behavior of a torsional pendulum because the thin drill pipe has a slender structure, which can be characterized by a constant stiffness. The bottom hole assembly is composed of thicker-walled tubes and heavier collars with negligible stiffness, for which the pipes behave as torsional springs and the collars behave as a rigid body. We suppose that a DC top drive rotates the system, and the only interaction of this system (rotary drilling system) with the formation is at the PDC drill bit. The proposed drilling system model in this study was developed under the SimScape/Matlab environment, where the drill string model is based on the fragmentation of all the drill pipes; each fragment represents a torsion pendulum subjected at their ends to viscous and/or dry friction [32]. The pendulum fragments are attached in series; thus, they are constructed altogether with the drill string [27]. The top drive is located at the upper end of the drill string as shown in Figure 1, while the lower end of the BHA is subjected to viscous friction and the Tob (torque on bit); its model is represented in Figure 2. The general model of the drill string is given in Equation (2), and its physical illustration is given in Figure 3 [29].

(2) $$\left\{\begin{array}{c} \dot{x}\left(t\right)=Ax\left(t\right)+B\Gamma \left(t\right)\\ y\left(t\right)=Cx\left(t\right) \end{array}\right.,$$

where $x\left(t\right)\in R^{4}$ is the state, $\Gamma \left(t\right)\in R^{2}$ is the input vector, and $y\left(t\right)\in R^{2}$ is the output vector with $x\left(t\right)={\left[\begin{array}{cccc} \phi & {\dot{\varphi }}_{t} & {\dot{\varphi }}_{b} & I \end{array}\right]}^{T}$, $\Gamma \left(t\right)={\left[\begin{array}{cc} T_{ob} & U \end{array}\right]}^{T}$, and $y\left(t\right)={\left[\begin{array}{cc} {\dot{\varphi }}_{t} & I \end{array}\right]}^{T}$.

The torque on the bit $T_{ob}\left({\dot{\varphi }}_{b},W_{ob}\right)$ constitutes an unknown entity to be estimated by an observer, and its influence on the dynamics of the BHA is reduced on the basis of the LQG controller [33]. Since the main purpose of this work is to provide a robust observer-based controller, if the mathematical model is not accurate enough, we can approach its real dynamics through well-designed observer feedback. To ensure the reliability of the drill string model, its open-loop responses were carefully studied and tested, as explained in the next subsection.

### 2.3. Model Responses

**Figure 4:**
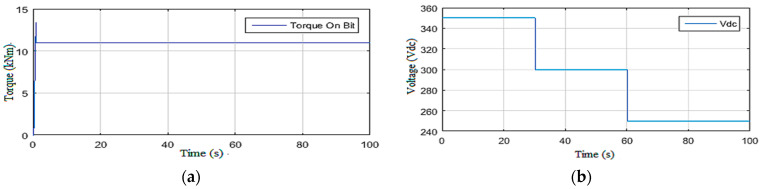
Inputs of Scenario 1: (**a**) the Tob; (**b**) the top drive voltage.

**Figure 5:**
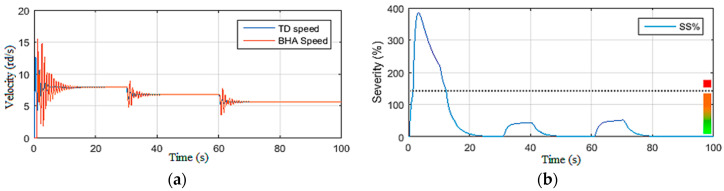
Outputs of Scenario 1**:** (**a**) drill bit speed; (**b**) stick-slip severity.

**Figure 6:**
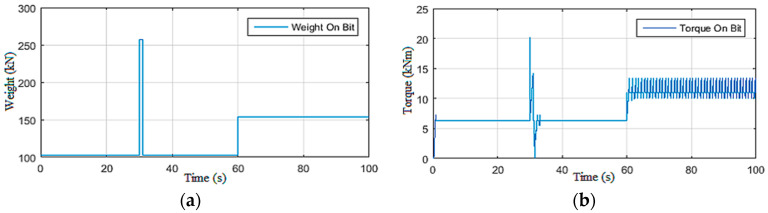
Inputs of Scenario 2: (**a**) weight on bit; (**b**) torque on bit (Tob).

**Figure 7:**
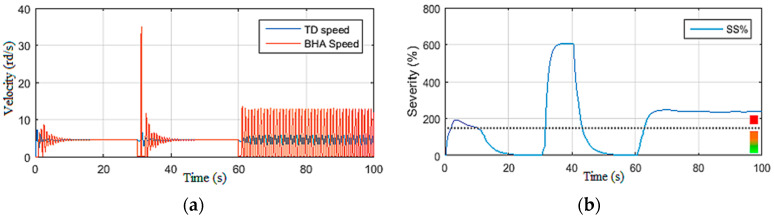
Outputs of Scenario 2: (**a**) drill bit velocity; (**b**) stick-slip severity.

The drill string simulations presented in this section were carried out to quantitatively approve the behavior of this model toward the observations and the recommendations provided by drillers to eliminate stick-slip vibration occurrences in the drill string [24]. On the basis of these recommendations, the simulation scenarios were conducted by varying one input at a time. First, the top drive supply voltage (*U*) was manipulated under constant Wob [19]. Then, in the second scenario, the Wob was changed while maintaining the voltage constant, as demonstrated in Figure 4, Figure 5, Figure 6 and Figure 7.

#### 2.3.1. Simulations under Constant Weight on Bit

In this scenario, the drill string system was driven by maintaining the Wob constant (154 kN ≈ 16 t) and varying the supply voltage of the top drive machine [19]. This supply voltage and the resistive torque acting on the drilling tool are illustrated in Figure 4, while the rotational speed and the severity of the torsional vibrations along the drill string are shown in Figure 5. It can be seen that, at the beginning, the vibrations were of level 3 (Table 1); then m at *t* = 13 s, the system became within the permissible vibration range (level 0) [24].

Despite the staircase descent of the top drive voltage of the power supply (up to 250 Vdc), the vibrations remained in the secure area where they were eliminated naturally without self-excitation [34], which is not possible in the field because the drilling system starts directly using a supply voltage of 250 Vdc due to energy consumption limitations [24].

#### 2.3.2. Simulations under Constant Voltage Vdc

In this second simulation scenario, the drill string system was driven by maintaining the supply voltage of the top drive machine constant (200 Vdc) while varying the Wob [19]. First, a static Wob was imposed on the bit, and then a disturbance was applied on the latter at *t* = 30 s as shown in Figure 6a. It can be noted that, directly after affecting the torque on the bit shown in Figure 6b, severe torsional vibrations occurred; they were of level 3 as shown in Figure 7a.

Moreover, these vibrations were excited by the increase in Wob at *t* = 60 s, and the drill string system underwent permanent stick-slip vibration of level 3, as demonstrated in Figure 7a,b. Such a situation can be very dangerous if not quickly detected and robustly controlled by an observer-based controller, as explained in the next section.

## 3. Observer-Based LQG Controller for Drill String

The observer-based controller presented in this section is a dynamic controller with feedback output and a two-stage structure [24,35]. First, the observer generates an estimate of the state variables of the system to be controlled, using the measured outputs of the speed and current of the top drive and the known inputs (voltage of top drive power supply) [36]. Second, the estimated state is treated as if it is equal to the exact state of the system, and it is consequently used by a static feedback controller to provide the controller law iteratively [33]. The control law and the estimated state are updated iteratively until the estimation error converges to a value less than the threshold error (in this study, *e =* 10^−3^).

### 3.1. Observer Synthesis

**Figure 8:**
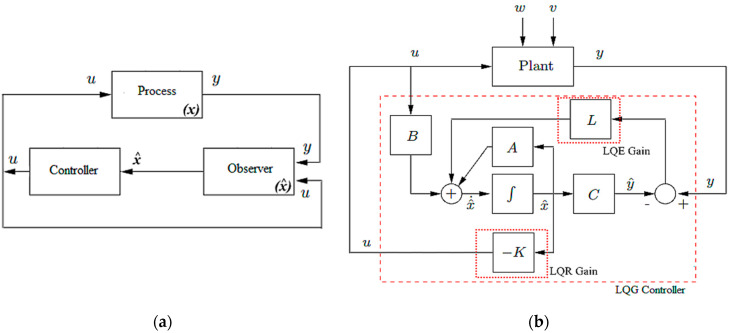
(**a**) Control strategy of the drill string in the rotary drilling system; (**b**) schematic diagram of designed observer-based LQG.

Observer-based controller synthesis for drill string requires a mathematical reformulation to be able to estimate the unknown input (Tob) and the design of a controller that follows a given drill bit speed reference, as highlighted in Figure 8a,b [36,37].

On the basis of the state model in Equation (2), an augmented model, where the unknown input (Tob) constitutes a new state variable (without dynamics), was formulated as given in Equation (3).

(3) $$\left\{\begin{array}{c} \left(\begin{array}{c} \dot{x}\left(t\right)\\ {\dot{T}}_{ob}\left(t\right) \end{array}\right)=\left(\begin{array}{cc} A & E\\ 0 & 0 \end{array}\right)\left(\begin{array}{c} x\left(t\right)\\ T_{ob}\left(t\right) \end{array}\right)+\left(\begin{array}{c} B_{2}\\ 0 \end{array}\right)u\left(t\right)+Mw\left(t\right)\\ y\left(t\right)=\left(\begin{array}{cc} c_{2} & 0 \end{array}\right)\left(\begin{array}{c} x\left(t\right)\\ T_{ob}\left(t\right) \end{array}\right)+v\left(t\right) \end{array}\right.,$$

$$A=\left(\begin{array}{cccc} 0 & 1 & -1 & 0\\ \frac{-k}{J_{t}} & \frac{-\left(c_{t}+c\right)}{J_{t}} & \frac{c}{J_{t}} & \frac{nK}{J_{t}}\\ \frac{k}{J_{b}} & \frac{c}{J_{b}} & \frac{-\left(c_{b}+c\right)}{J_{b}} & 0\\ 0 & \frac{-nK}{L} & 0 & \frac{-R}{L} \end{array}\right),\;E=\left(\begin{array}{c} 0\\ 0\\ \frac{-1}{J_{b}}\\ 0 \end{array}\right),\;B=\left(\begin{array}{c} 0\\ 0\\ 0\\ \frac{1}{L} \end{array}\right),\;c_{2}=\left(\begin{array}{cccc} 0 & 1 & 0 & 0\\ 0 & 0 & 0 & 1 \end{array}\right),$$

where *w*(*t*) and *v*(*t*) represent independent Gaussian white noises with covariance matrices *W = W_0_ ≥ 0* and *V = V > 0*, respectively. The observer for the rotary drilling system was then designed by choosing the following set values:

$$M=\left(\begin{array}{ccccc} 2\times 10^{2} & 0 & 0 & 0 & 0\\ 0 & 1 & 0 & 0 & 0\\ 0 & 0 & 1 & 0 & 0\\ 0 & 0 & 0 & 2\times 10^{2} & 0\\ 0 & 0 & 0 & 0 & 2\times 10^{2} \end{array}\right),V=\left(\begin{array}{cc} 1\times 10^{2} & 0\\ 0 & 1\times 10^{2} \end{array}\right)\;W=\left(\begin{array}{ccccc} 1 & 0 & 0 & 0 & 0\\ 0 & 1\times 10^{5} & 0 & 0 & 0\\ 0 & 0 & 1\times 10^{3} & 0 & 0\\ 0 & 0 & 0 & 1\times 10^{3} & 0\\ 0 & 0 & 0 & 0 & 1\times 10^{7} \end{array}\right),$$

along with the following inputs:

$$x_{z}\left(t\right)=\left(\begin{array}{c} x\left(t\right)\\ T_{ob}\left(t\right) \end{array}\right),A_{z}=\left(\begin{array}{cc} A & E\\ 0 & 0 \end{array}\right),\;B_{z}=\left(\begin{array}{c} B_{2}\\ 0 \end{array}\right),C_{z}=\left(\begin{array}{cc} c_{2} & 0 \end{array}\right).$$

The designed observer of the system (Equation (3)) that minimizes the estimation error covariance at the steady state is given by Equation (4).

(4) $${\dot{\hat{x}}}_{z}\left(t\right)=\left(A_{z}-L_{k}C_{z}\right){\hat{x}}_{z}\left(t\right)+B_{z}u\left(t\right)+L_{k}y\left(t\right),$$

where *L* is the observer gain given in Equation (5) [33,36].

(5) $$L_{k}=Y^{*}C_{z}^{T}V^{-1}.$$

$Y^{*}$ is the solution of the *Riccati* Equation (6) [24].

(6) $$YA_{z}^{T}+A_{z}Y-YC_{z}^{T}V^{-1}C_{z}Y+MWM^{T}=0.$$

### 3.2. Controller Synthesis

The designed controller is based on LQG in the context of a servomechanism [17]. For this reason, a mathematical reformulation of the state model Equation (3) is given in Equation (7) [37].

(7) $$\left\{\begin{array}{c} \left(\begin{array}{c} \dot{x}\left(t\right)\\ \dot{\zeta }\left(t\right) \end{array}\right)=\left(\begin{array}{cc} A & 0\\ -\alpha c_{r} & 0 \end{array}\right)\left(\begin{array}{c} x\left(t\right)\\ \zeta \left(t\right) \end{array}\right)+\left(\begin{array}{c} B_{2}\\ 0 \end{array}\right)u\left(t\right)+\left(\begin{array}{c} 0\\ \alpha \end{array}\right)y_{r}\left(t\right)\\ y\left(t\right)=\left(\begin{array}{c} x\left(t\right)\\ \zeta \left(t\right) \end{array}\right) \end{array}\right.,$$

where *ξ* is the new state variable that represents the integration of the tracking error, *y_r_* is the reference for the rotational speed of the drill bit, and *c_r_* = (0 0 1 0) is a controller setting parameter with *α =* 3 [26].

By putting $x_{q}\left(t\right)=\left(\begin{array}{c} x\left(t\right)\\ \zeta \left(t\right) \end{array}\right)$, $A_{q}=\left(\begin{array}{cc} A & 0\\ -\alpha c_{r} & 0 \end{array}\right)$, $B_{q}=\left(\begin{array}{c} B_{2}\\ 0 \end{array}\right)$, the LQR control $u\left(t\right)$ that minimizes the cost function $J=\int_{0}^{\infty }\left(x_{q}^{T}Qx_{q}+u^{T}Ru\right)dt$ and ensures the tracking of the reference $y_{r}$ is obtained in Equations (8) and (9).

(8) $$u\left(t\right)=F_{k}x_{q}\left(t\right),$$

(9) $$F_{k}=R^{-1}B_{q}^{T}X^{*},$$

where $X^{*}$ is the solution of *Riccati* Equation (10) [24].

(10) $$XA_{q}+A_{q}^{T}X-XB_{q}R^{-1}B_{q}^{T}X+Q=0,$$

with

$$Q=\left(\begin{array}{ccccc} 1 & 0 & 0 & 0 & 0\\ 0 & 1 & 0 & 0 & 0\\ 0 & 0 & 1\times 10^{3} & 0 & 0\\ 0 & 0 & 0 & 1\times 10^{1} & 0\\ 0 & 0 & 0 & 0 & 1\times 10^{5} \end{array}\right),R=1\times 10^{-3}.$$

## 4. Results and Discussion

**Figure 9:**
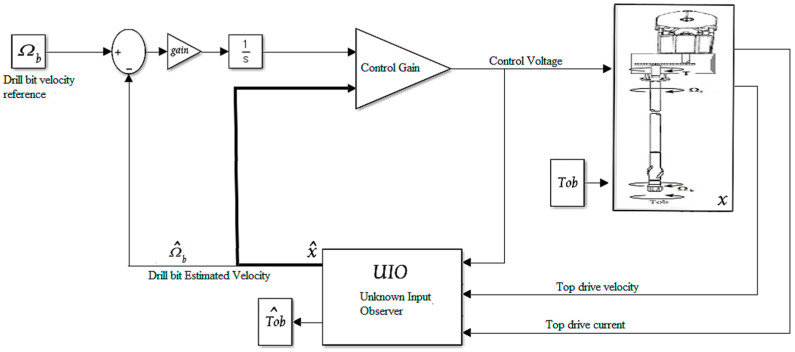
LQG observer-based controller block for drill string system.

To test the performance of the designed observer-based LQG controller shown in Figure 9, several simulation scenarios were studied [33]. First, the accuracy of the designed observer without any control was investigated; then, the whole observer–controller system was examined to its limits [36]. The parameters of the moments of inertia, the stiffness of the drill pipe, and the viscous friction coefficients were all calculated from basic equations by taking the drilling parameters of an operating rig, namely, the length of the drill pipes and drill collars, the diameter and thickness of drill string, and the mud viscosity [26].

### 4.1. Observer Performance Tests

In these tests, the observer simulations were conducted by disabling the controller and imposing several scenarios by varying each time one input variable among the supply voltage (*U*), weight on the bit (Wob), disturbed measurements, and parametric variation on the drill string model [26].

#### 4.1.1. Constant Wob with U Step

**Figure 10:**
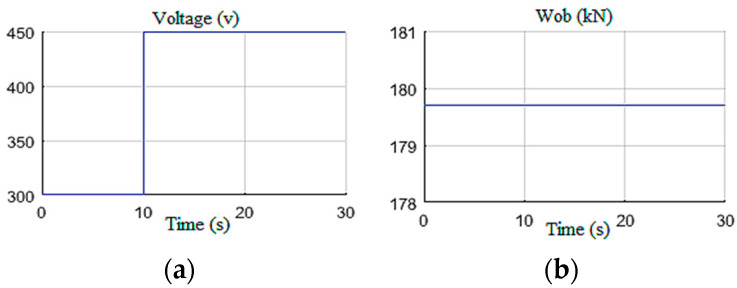
Test inputs for Scenario 1: (**a**) the voltage; (**b**) the Wob.

**Figure 11:**
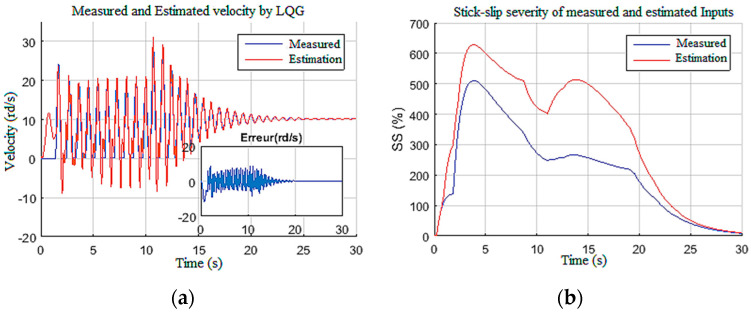
Test outputs for Scenario 1: (**a**) drill bit velocity; (**b**) severity.

A constant weight was applied to the drill bit equivalent to 70% of the overall weight of the drill string, which is about 29 tons. The supply voltage was increased stepwise from 300 V to 450 V at *t* = 10 s as shown in Figure 10. These input settings induced stick-slip vibrations along the drill string during the first 10 s; then, by increasing the top drive velocity, the vibrations were gradually eliminated after 12 s, as shown in Figure 11a. This figure shows the measured and estimated speed of the drilling tool and their corresponding estimation errors [38]. It can be noted that the observer provided a good estimation with zero mean errors and tolerable variance during stick-slip and during manual vibration suppression [16].

**Figure 12:**
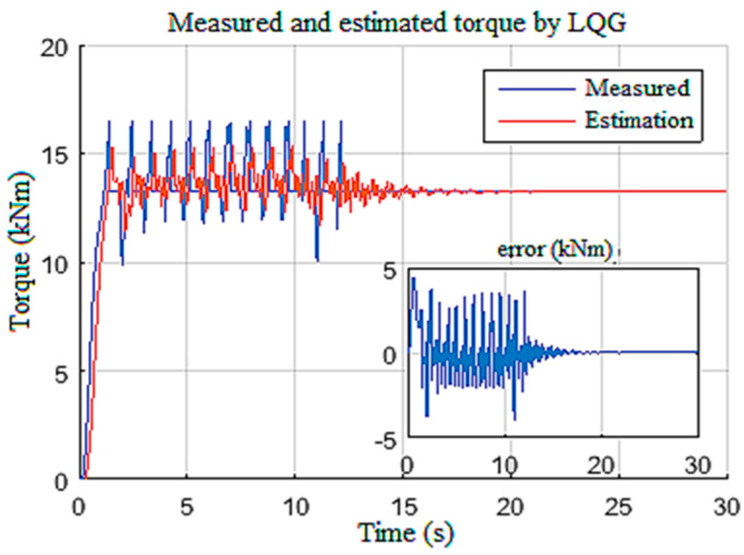
Torque on bit response for Scenario 1.

**Figure 13:**
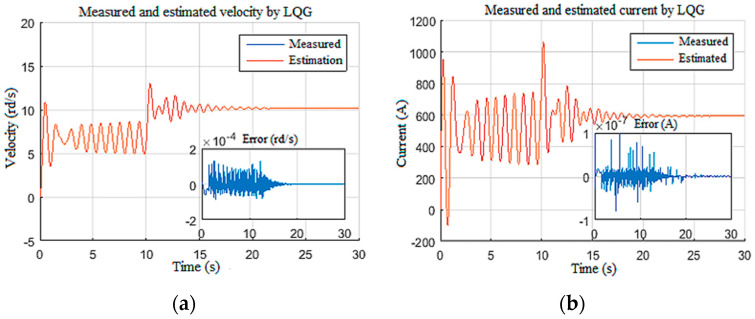
Top drive responses for Scenario 1: (**a**) velocity; (**b**) current.

Moreover, this estimation provided an efficient way of classifying the stick-slip severity (*SS%*) of the drill string, as given in Figure 11b. In addition, the designed observer estimated the unknown input Tob (Figure 12), which can manifest as an internal disturbance on the drill string model [33]. Measurable states of the system, namely, the speed of the top drive and the armature current, were also estimated with good accuracy by the observer, as demonstrated in Figure 13.

#### 4.1.2. Random Wob with Constant U

**Figure 14:**
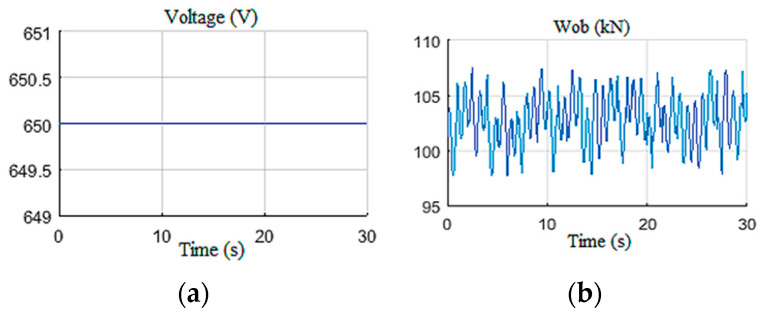
The input of the observer in Scenario 2: (**a**) voltage; (**b**) weight on bit.

**Figure 15:**
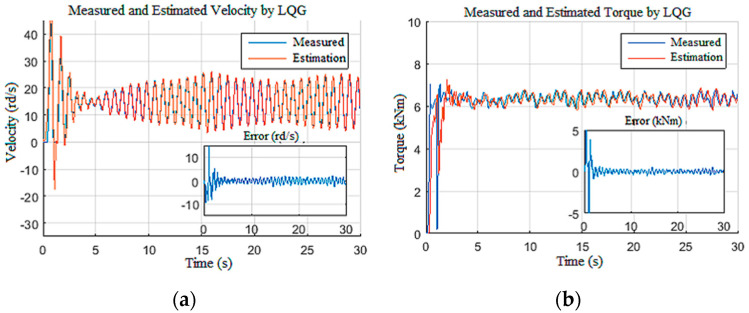
Outputs of the observer in Scenario 2: (**a**) drill bit velocity; (**b**) Tob.

In this scenario, the behavior of the designed observer toward a random Wob variation was tested [19]. This variation is practically justified by the simulation of the dynamic axis of the drill string [11]. The inputs of the observer are shown in Figure 14, while the outputs are shown in Figure 15. It was noticed that the observer afforded acceptable drill bit velocity and *Tob* estimations [33].

**Figure 16:**
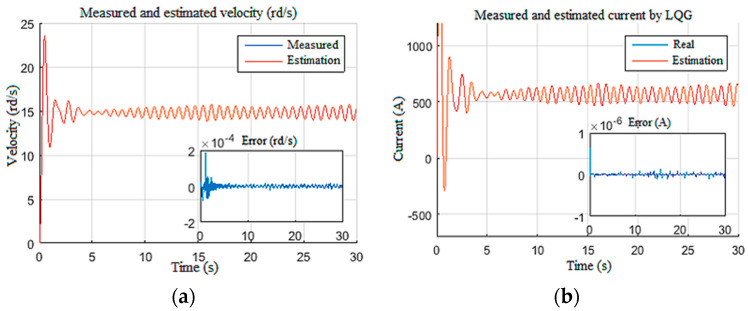
Top drive responses for Scenario 2: (**a**) velocity; (**b**) current.

Furthermore, the inputs created fluctuation in the top drive speed around 15 rd/s (Figure 16a) and an armature current around 500 A (Figure 16b) with a large starting current peak due to controller deactivation [24]. The speed and the current fluctuations were estimated with good accuracy even with the presence of an unknown input of random dynamics (Wob).

#### 4.1.3. Disturbed Measurements

**Figure 17:**
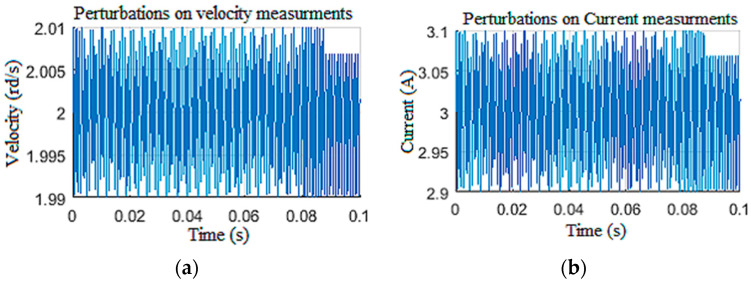
Perturbations in the measurement: (**a**) velocity; (**b**) current.

**Figure 18:**
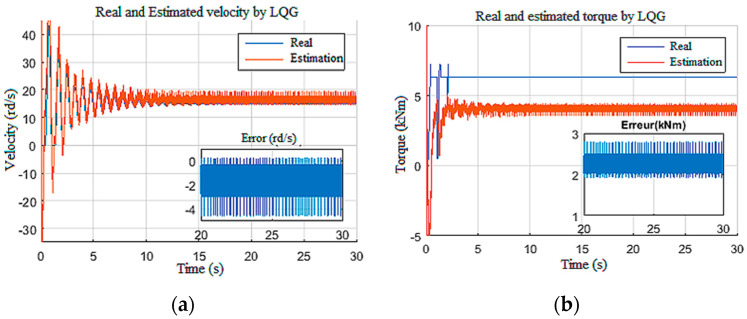
Responses for Scenario 3: (**a**) drill bit velocity; (**b**) torque on bit.

**Figure 19:**
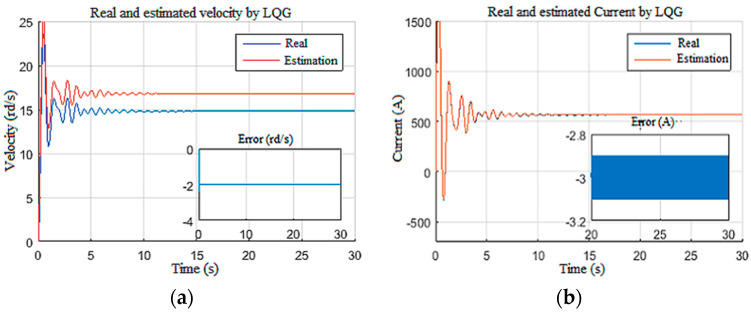
Top drive responses for Scenario 3: (**a**) velocity; (**b**) current.

In this scenario, the model was simulated with a constant tension of 650 V for the power supply and a constant Wob of 103 kN. To test the robustness of the observers against disturbances on the measurements [12], the speed and the current measurements were corrupted by unstructured disturbances of 1 kHz frequency as given in Figure 17. The structured perturbations were generally characterized by a Gaussian distribution in the frequency domain, while the unstructured perturbations had random distribution laws. As expected, the LQG observer did not provide good filtering of the DC component of biased disturbances, since the perturbations were unstructured [35]. This limitation had a direct influence on the unknown input estimation, as shown in Figure 18, as well as the known input estimation (Figure 19). Henceforth, it was assumed that the drill string system should be under structured perturbation in the rest of this study.

#### 4.1.4. Parametric Variation

**Figure 20:**
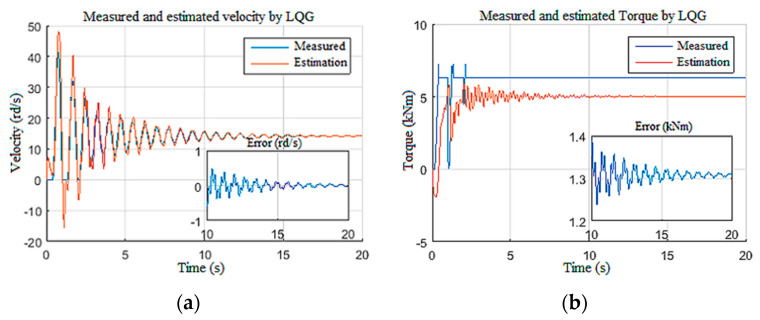
Outputs of LQG from Scenario 4: (**a**) drill bit velocity; (**b**) Tob.

In this test, the system was under the same input setting of Scenario 3: a constant supply voltage of 650 V and a constant Wob of 103 kN. The parameters of the drill string, namely, the resistance (*r*), the inductance (*l*), the length, and the mass of the string, were deviated, and these parameters were used for the calculation of the moments of inertia and the viscous friction coefficients [38]. Then, it can be noted that the designed observer provided good robustness (Figure 20a) [29]. However, they exhibited sensitivity to changes in the torque constant (*K*) [26]. Figure 20b shows the simulation results with a deviation of 3% on the nominal value of the torque constant (*K*) [24].

### 4.2. Observer-Based Controller’s Performance Tests

In this subsection, the full observer-based controller performances were tested, whereby the controller robustness depends strongly on the accuracy of the estimated states provided by the observer [29]. The primary goal of the designed control system is to establish a predefined dynamic on the top drive in such a way that the drill bit pursues the convenient speed reference, which consequently mitigates the torsional vibrations and suppresses levels 2 and 3 of stick-slip vibration in the drill string in the shortest possible time [16].

#### 4.2.1. Reference Tracking

**Figure 21:**
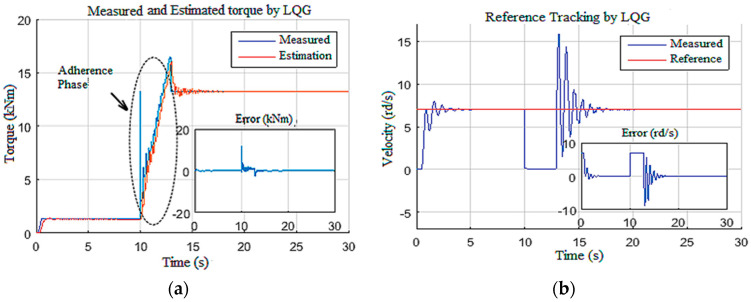
LQG observer-based controller responses: (**a**) Tob; (**b**) bit velocity.

**Figure 22:**
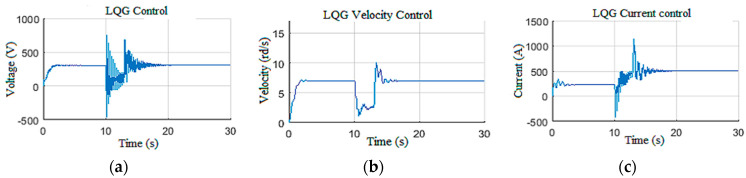
Controller responses: (**a**) voltage; (**b**) velocity; (**c**) current.

In this scenario, the control system was subjected to an echelon Wob input designed to test the controller robustness against the occurrence of an unknown amplitude disturbance. The simulation was initiated with a low Wob (25 kN) and a reference of *7* rd/s. Then, at *t =* 10 s, the Wob was increased to 180 kN, and the Tob generated by this increase is shown in Figure 21a. This increase caused an abrupt stop of the drill bit, as demonstrated in Figure 21b. The designed LQG controller forced the drill bit to leave this stuck phase at *t =* 14 s and reached the desired speed reference again within 4 s (Figure 21b); this action controlled the top drive with tolerable speed and current states, as demonstrated in Figure 22.

#### 4.2.2. Stick-Slip Suppression

**Figure 23:**
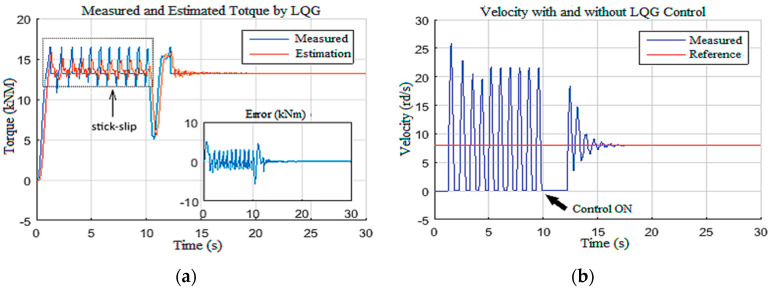
Output response for stick-slip suppression: (**a**) Tob; (**b**) bit velocity.

In this scenario, the observer-based LQG controller performances are discussed for stick-slip vibration suppression with high severity (level 2 and level 3) [38,39]. To do so, the drill string model was firstly driven without any controller and with Wob = 180 kN and voltage equal to 300 V; these input set values induced severe stick-slip vibrations along the drill string, as shown in Figure 23. At *t* = 10 s, the designed control system was activated. It can be noted that the severe stick-slip vibrations were suppressed by the LQG system within 3 s.

**Figure 24:**
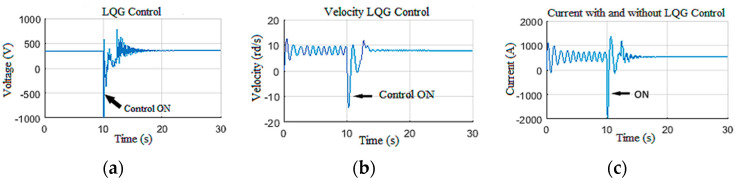
Top drive response for stick-slip suppression: (**a**) voltage; (**b**) velocity; (**c**) current.

Thus, it can be concluded that the designed observer-based controller demonstrated high robustness toward levels 2 and 3 of stick-slip vibrations [33]. The surface parameters and their estimation are presented in Figure 24.

#### 4.2.3. Structured Disturbance Filtering

**Figure 25:**
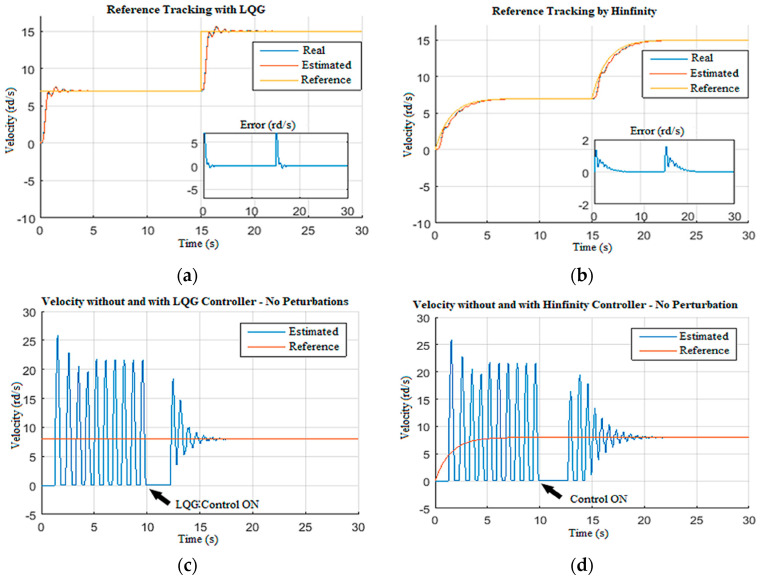

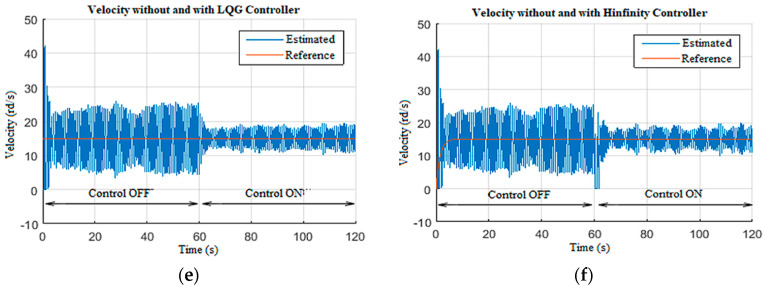
Comparison between LQG and H∞ responses: (**a**) reference tracking with LQG; (**b**) reference tracking with H∞; (**c**) drill bit velocity by LQG without disturbances; (**d**) drill bit velocity by H∞ without disturbances; (**e**) drill bit velocity by LQG under structured disturbances; (**f**) drill bit velocity by H∞ under structured disturbances.

In this simulation scenario, the ability of the designed observer-based controller in reducing the structured perturbations on the Wob is demonstrated [19,40]. These perturbations are generally caused by the drill string axial dynamics [4]. To do so, the system was driven without control under constant Wob and voltage with structured perturbations; then, at *t =* 60 s, the designed control system was activated, as shown in Figure 25. Figure 25a,b show the reference tracking responses for LQG and H∞, respectively. Even though the fluctuations were less in the H∞ curve (Figure 25b), the response time for LQG is shorter, which indicates better robustness. Figure 25c,d show the estimated drill bit velocity without structured perturbation for LQG and H∞, respectively. The LQG observer-based controller suppressed the high-frequency stick-slip vibration in 5 s (Figure 25c), while the H∞ observer-based controller took 8 s (Figure 25d). Then, structured perturbations were introduced to the Wob input in order to compare the performances of LQG and H∞, as shown in Figure 25e,f, respectively.

**Figure 26:**
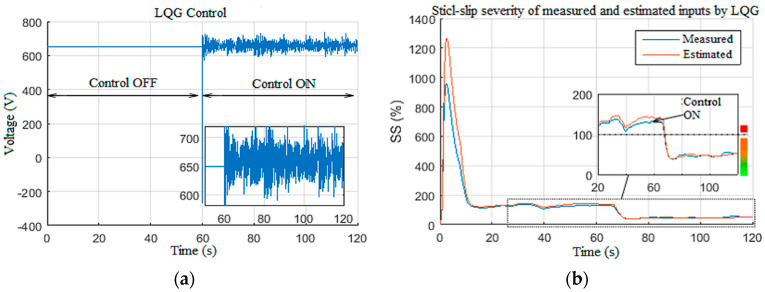
LQG response under structured disturbances: (**a**) LQG control voltage; (**b**) stick-slip severity.

The LQG observer-based controller in Figure 25e showed a slight improvement in structured perturbation rejection compared to H∞ in Figure 25f. The LQG responses presented in Figure 25 demonstrate the robustness of the designed observer-based controller in filtering the structured perturbation that appeared in the Wob and suppressing the stick-slip vibrations under such conditions; it was also deduced that the transition in the LQG control (Figure 26a) was smoother in comparison to H∞ controller developed in [24,41]. Thus, it was proven that LQG observer-based controller provided better results than H∞ under structured perturbations that appear often during drilling; the proposed LQG took 3 s to attenuate the high-frequency stick-slip severity from high (level 3) to low or very low (levels 1 and 0), as shown in Figure 26b.

## 5. Performance Limits

**Figure 27:**
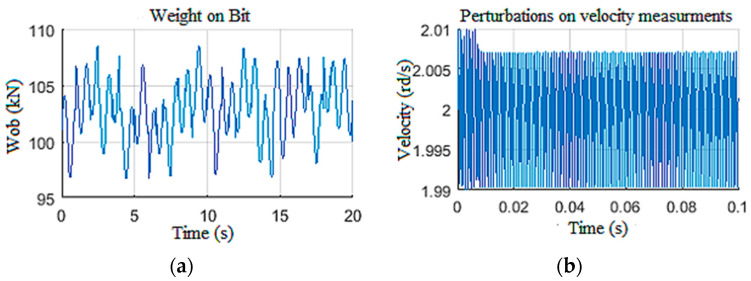
Disturbances on LQG inputs: (**a**) Wob; (**b**) induced perturbations.

In this section, we pushed the controller to its limits by imposing disturbances on the Wob and the velocity measurements (Figure 27a,b), while imposing parametric variations along the length of drill pipes and drill collars [26]. This variation was on the order of 130% of the nominal value, in addition to a torque constant variation on the order of 103%.

**Figure 28:**
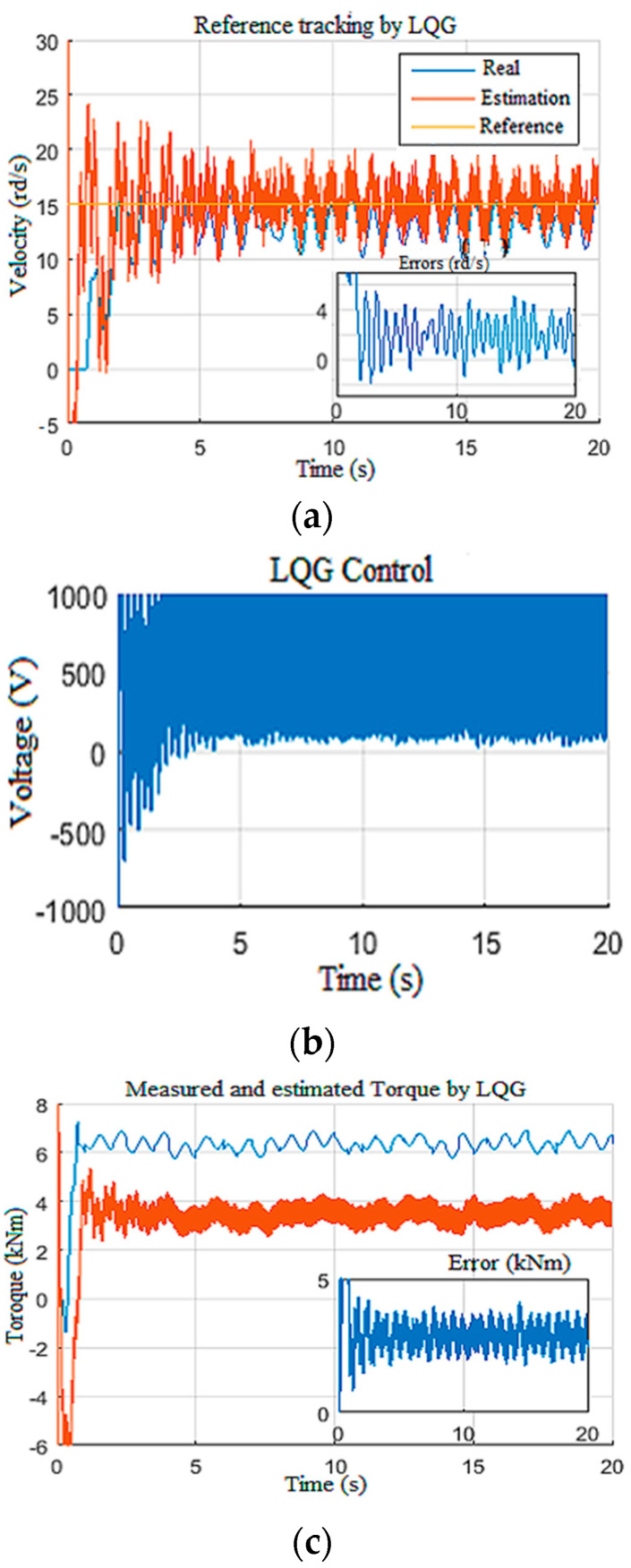
Extreme limitation of LQG responses: (**a**) drill bit velocity; (**b**) voltage; (**c**) the Tob.

**Figure 29:**
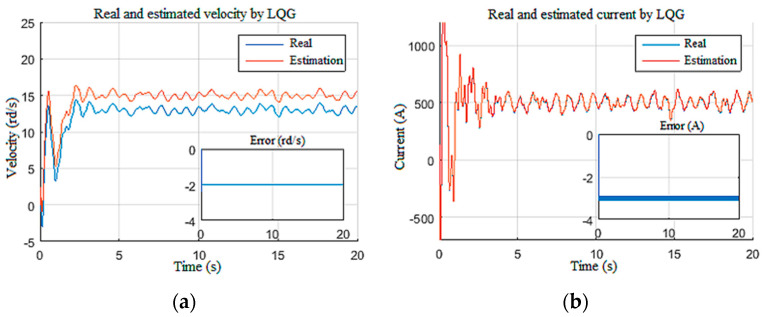
Performance limitation of LQG top drive responses: (**a**) velocity; (**b**) current.

Figure 28 demonstrates that the observer-based LQG controller generated a static tracking error of 2 rd/s with a poorly estimated drill bit velocity (Figure 28a). From Figure 28b, we can notice that the LQG control law presented saturation on the provided voltage to the top drive, and the Tob estimation was not enough (Figure 28c) [32]. Moreover, in this extreme scenario, even the known surface parameter estimations suffered from the static error of −2 rd/s for velocity and *−*3 A for the current, as shown in Figure 29a,b, respectively.

## 6. Conclusions

The main objective of this paper was to develop an observer-based control system that can suppress the severe stick-slip vibrations along the drill string of the rotary drilling system. An observer was firstly designed to estimate the unknown drill bit velocity, as well as the Tob, and then a controller was designed in a two-stage structure to suppress the vibrations on the basis of their quantitative severity levels. The main contribution of this study is the proposition of the observer-based LQG controller as a robust strategy to suppress level 2 and level 3 stick-slip vibrations. Several simulation scenarios were conducted to investigate and highlight the effectiveness of the proposed approach. The obtained results confirmed the improved performances and the good robustness of the observer-based controller, for which the stick-slip vibrations were suppressed within 3 s. This is much better than the manual practices currently used in drilling fields. Furthermore, the LQG control response was smoother than the H∞ response for structured perturbation; however, for unstructured perturbations, it did not provide any improvement. Thus, it is highly recommended to consider the proposed approach in suppressing the stick-slip vibrations in the case of real-time MWD tools unavailability, as well as in investigating less sensitive estimation techniques to the unstructured perturbations if there are any in the operating rotary drilling systems.

## Figures and Tables

**Table 1:** Stick-slip severity level classification [24].

Stick-Slip Severity Level	Classification	SS%
0	Very low	0 to 50%
1	Low	50% to 100%
2	Mean	100 to 150%
3	High	150 to 200%
